# Quackery, and the National System of Medical Education

**Published:** 1871-09

**Authors:** H. R. Hopkins

**Affiliations:** Buffalo


### Correspondence.

*To the Editor of the Buffalo Medical and Surgical Journal.*

In my last letter I referred to the disgrace brought upon the profession by the low standard of medical education. To-day I would charge the responsibility of the existence and flourishing condition of the multitudinous forms of quackery upon our national system of medical education. I am well aware that it has long been the habit of members of our profession, whose positions entitle their opinions to great weight, upon being asked: Why is it that so many irregular practitioners of medicine meet with great pecuniary success? and, why are so many different patent medicines yearly sold at an enormous profit to the manufacturer, and consumed at an equal disadvantage to the consumer? To answer that the fools are not all dead yet. Can the profession thus lightly wash its hands of this matter? Is not the standard of the professed attainment *at fault?*

Is it not a fact that from a profusion of private corporations, endowed with the authority to confer the degree of Doctor in Medicine, whose controlling parties have a pecuniary interest in the number of degrees thus conferred; there results a profession which actual experience has proven to be little better than the quacks. Not that professional ability is of less value, but that professional responsibilities are often entrusted to men who are neither capable by virtue of literary cultivation, professional attainment, or moral worth, to do anything which can increase the respectability of the profession. Upon our system of medical education rests the responsibility that the degree of Doctor of Medicine no longer entitles its holder to the respect due to a gentleman of professional culture, and high moral character; but only entitles him to the legal right to practice medicine.

At the same door lies the responsibility that throughout our whole country there is scarcely an organization of medical men whose real object is the promotion of true science, and a knowledge of the healing art; but of organizations for defence, for aggression, and for mutual admiration, there are multitudes. Is it because the duties and responsibilities of a Physician are unimportant that the guarantees for protecting the profession from those who are attracted to its ranks from cupidity, are almost entirely disregarded? To none of the honorable profession, and to few of the lucrative trades are there such sluice-ways; and yet, no man in the community, neither the man of letters, the lawyer, nor the divine, has such opportunities for usefulness, and wields such influence as the Physician, provided he is fitted by nature and cultivation for his vocation. To prove that the pitiful condition of the profession in this country is owing to our national system of medical education, we have only to turn our attention to those countries in which one must show himself to have professional ability of "what would be with us a marvelously high standard" before he is entrusted with the responsibility of the treatment of disease, or the custody of health. For example, the degree of Doctor in Medicine in Germany represents fourteen years continuous application, under teachers who are specially trained for their work. With us, it represents nothing but that the holder has parted with two or three hundred dollars to some one of our numerous medical schools, and the result is what any one might expect. At home they enjoy the universal respect and confidence of their constituents, and are everywhere known to lead the world; while we are working hard to keep even with quacks and patent medicines in the confidence of our people, and abroad, our degrees, with but few exceptions, are not even recognized.

For years the cry has come up from our Medical Associations, deprecating the low standard of medical education. You can scarcely look through a Medical journal, but you find the subject dwelt upon, either in editorial or correspondence. It is to Medical journalists and Medical Associations, a subject which can be discussed and resolved upon, when nothing else can be thought of, and yet, with the exception of the reformation lately taken place in the Medical Department of Harvard University, not one thing has been done, either by medical association or medical school, to bring about the desired reform.

Our National and State Medical Associations meet, resolve, and re-resolve from year to year; but the cancer still eats away the respectability, integrity, and honor of the profession. It is most humiliating, to think that our profession is so markedly behind our country and the age.

Recognizing the importance of reforming this evil, to what body shall we look for assistance? In the County Medical Societies is the power to work this reformation. This organization is the only one created by law for the purpose of "promoting true science, and a knowledge of the healing art." The County Medical Society is the only guardian of the profession from ignorance and cupidity. The laws of our country regulating the practice of Physic and Surgery, although very lax when compared with those of England and the countries of Continental Europe, define with exactness the requirements for a legally practicing physician. It is the duty of the County Medical Societies to see that these laws are enforced, and it is in the power of the same Societies to secure the enactment of other laws, when experience has proven that those we now have are insufficient. What account these Societies can give of their stewardship, is too well known. They have slept while standing guard.

The law recognizes three epochs in the preparatory life of the physician; two of these, the first and the last, are under the control of County Medical Societies. First, they must pass judgment upon the literary attainments and moral character of the young man who desires to become a student of medicine before he can enter upon such study. What would have been the effect upon the profession if proper advantage had been taken of this opportunity for raising the standard of medical education can hardly be overestimated. Again, the County Society is called upon to decide whether the person holding a degree of Doctor in Medicine, and who wishes to connect himself with such Society, and thus become a legally practicing physician, is "of temperate habits, good moral character, and legally authorized to practice." What a spectacle we present when we entirely ignore these opportunities for raising both the standard of professional attainment, and professional honor, and then cross the continent to attend a National Medical

Association, and there resolve that "we deplore the low standard of medical education."

Reason points, and experience proves, that assistance in this work can neither come from medical schools or medical associations, controlled by the faculties of such schools. Evolution has not yet carried human nature to that point of perfection justifying the expectation of man's doing without pressure, that which he sees is against his interests.

Unfortunate it has been for the medical profession, and unfortunate it will be for our national system of medical education, that the good of the one is not the good of the other.

H. R. HOPKINS.

Buffalo, September, 1871.

